# Artificial Intelligence in Infectious Disease Diagnostic Technologies

**DOI:** 10.3390/diagnostics15202602

**Published:** 2025-10-15

**Authors:** Chao Dong, Yujing Liu, Jiaqi Nie, Xinhao Zhang, Fei Yu, Yongfei Zhou

**Affiliations:** 1 Hebei Key Laboratory of Analysis and Control of Zoonotic Pathogenic Microorganism, College of Life Sciences, Hebei Agricultural University, Baoding 071001, China; 15613282208@163.com (C.D.);; 2 Institute of Hemu Biotechnology, Beijing Hemu Biotechnology Co., Ltd., Beijing 102206, China

**Keywords:** AI, infectious disease diagnostics, HTS, medical imaging, POCT, deep learning, machine learning

## Abstract

Artificial intelligence (AI), as an emerging interdisciplinary field dedicated to simulating and extending human intelligence, is increasingly integrating into the domain of infectious disease medicine with unprecedented depth and breadth. This narrative review is based on a systematic literature search in databases such as PubMed and Web of Science for relevant studies published between 2018 and 2025, with the aim of synthesizing the current landscape. It demonstrates transformative potential, particularly in the realm of diagnostic assistance. Confronting global challenges such as pandemic control, emerging infectious diseases, and antimicrobial resistance, AI technologies offer innovative solutions to these pressing issues. Leveraging its robust capabilities in data mining, pattern recognition, and predictive analytics, AI enhances diagnostic efficiency and accuracy, enables real-time monitoring, and facilitates the early detection and intervention of outbreaks. This narrative review systematically examines the application scenarios of AI within infectious disease diagnostics, based on an analysis of recent literature. It highlights significant technological advances and demonstrated practical outcomes related to high-throughput sequencing (HTS) for pathogen surveillance, AI-driven analysis of digital and radiological images, and AI-enhanced point-of-care testing (POCT). Simultaneously, the review critically analyzes the key challenges and limitations hindering the clinical translation of current AI-based diagnostic technologies. These obstacles include data scarcity and quality constraints, limitations in model generalizability, economic and administrative burdens, as well as regulatory and integration barriers. By synthesizing existing research findings and cataloging essential data resources, this review aims to establish a valuable reference framework to guide future in-depth research, from model development and data sourcing to clinical validation and standardization of AI-assisted infectious disease diagnostics.

## 1. Introduction

With the rapid advancement of information technology, artificial intelligence (AI) is driving transformative changes in the healthcare sector by leveraging its unparalleled capabilities in data processing, pattern recognition, and intelligent decision-making [1,2,3]. The diagnosis of infectious diseases continues to face significant challenges, including the rapid emergence of novel pathogens, the complexity of differential diagnosis, and the urgent need for speed in outbreak containment [4,5]. By simulating core aspects of human cognition, AI technology enables in-depth mining and precise interpretation of medical and microbiological data, providing unprecedented perspectives and powerful analytical tools for accurate pathogen identification, antibiotic resistance prediction, and epidemic trend assessment [6,7,8]. Amidst the global maldistribution of medical resources, the pressing need to enhance diagnostic accuracy for complex infections, and escalating pandemic threats, AI-assisted diagnostic technology demonstrates considerable value and transformative potential [9]. These applications span critical areas: from the rapid identification of pathogens via genomic sequencing to the automated analysis of medical imaging; and from intelligent POCT systems to real-time epidemic surveillance networks [10,11,12]. Consequently, AI has transcended traditional diagnostic paradigms by enhancing data processing efficiency and recognizing complex patterns. Its unique advantages offer promising solutions to persistent clinical challenges, propelling infectious disease diagnosis toward a more efficient, precise, and proactive future [13,14,15].

The application of artificial intelligence in infectious disease diagnostics has gained considerable scholarly attention in recent years, with a growing body of review literature contributing to the field. While previous reviews have often provided in-depth examinations of specific technological domains—such as medical imaging analysis or high-throughput sequencing—and established a solid knowledge base, there remains a need for comprehensive analyses that integrate multiple technological approaches. This review establishes a systematic framework that brings together three key technological directions—high-throughput sequencing, medical imaging, and point-of-care testing—and introduces a dedicated new section on Available Databases for AI-Driven Infectious Disease Diagnostics. This section surveys crucial public data resources spanning genomics, medical imaging, and clinical records, which are foundational for training and validating robust AI models. Alongside a synthesis of technological developments and these data resources, this review systematically addresses critical challenges in clinical translation—such as data quality, model generalizability, economic viability, and regulatory considerations. It is hoped that this work will provide researchers with a structured and insightful reference that balances breadth with critical depth.

## 2. AI in High-Throughput Sequencing for Pathogen Surveillance

The integration of real-time genomic and epidemiological surveillance is crucial for the rapid diagnosis, tracking, and control of infectious disease outbreaks. By enabling rapid analysis of massive sequencing datasets, AI technology facilitates pathogen identification, variant monitoring, and outbreak investigation, thereby transforming this field.

### 2.1. Pathogen Identification and Characterization

AI plays a critical role in high-throughput sequencing (HTS)-driven pathogen research, demonstrating particular strength in pathogen identification, drug resistance analysis, and quality control of sequencing data [16,17]. For instance, Christians et al. established an AI-driven statistical model for antimicrobial resistance (AMR) that accurately traces detected resistance genes (such as mecA, vanA, and blaCTX-M) to specific bacterial pathogens using probabilistic inference, thereby enabling culture-independent prediction of phenotypic resistance. This study also extensively incorporated in silico validation methods, demonstrating the utility and reliability of AI in the high-throughput evaluation of detection limits, as well as primer inclusivity and exclusivity [18].

Furthermore, the AI model developed by Lin et al., which utilizes a Ct-guided nine-state Markov process based on viral Ct values, illustrates how temporal biological data can be leveraged to accurately quantify infection dynamics. Capable of reconstructing full viral kinetic trajectories from incomplete real-world observations, this model has been used to characterize transmission patterns of the Alpha and Omicron variants, estimating key epidemiological parameters such as the basic reproduction number and generation interval. It has also informed the design of targeted containment strategies. This methodology extracts meaningful patterns from high-dimensional temporal data to support pathogen surveillance and decision-making, offering a valuable framework for utilizing HTS data in pathogen identification, variant monitoring, and transmissibility assessment [19].

**Figure 1:**
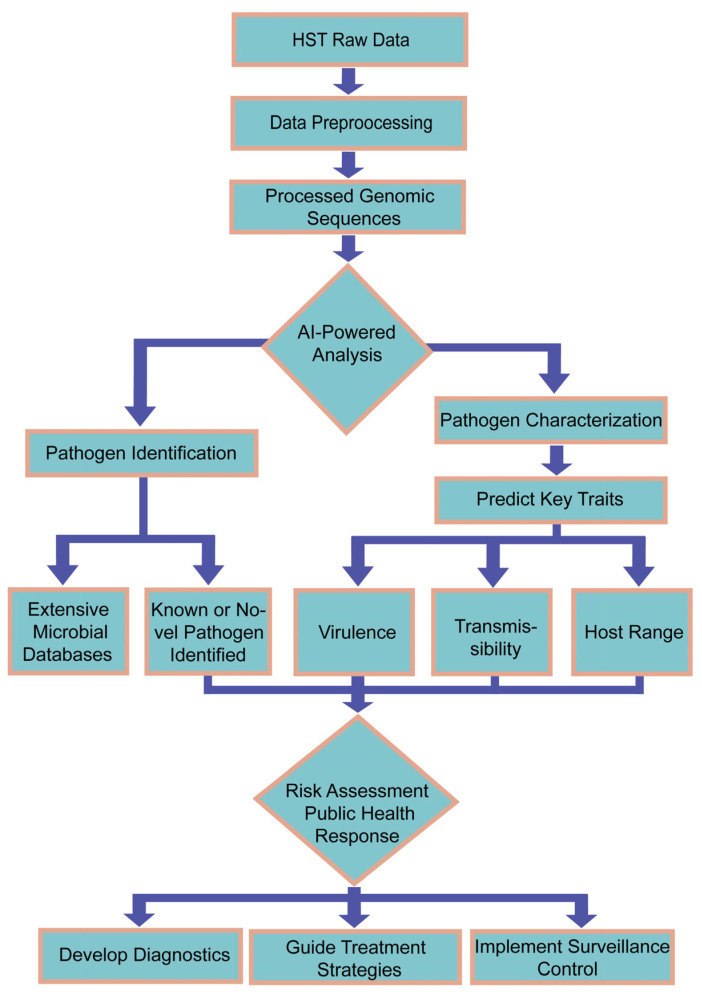
AI-Powered High-Throughput Pathogen Characterization and Response Framework.

Machine learning demonstrates extensive application prospects in pathogen classification/identification and multi-omics integration analysis. For example, support vector machines (SVM), random forest algorithms, and k-mer-based analytical methods enable the efficient classification and identification of microbial gene sequences (such as 16S rRNA), thereby facilitating the rapid detection of pathogens including bacteria and viruses. Moreover, artificial intelligence enables the integrative analysis of multi-omics data (spanning genomic, metabolomic, and transcriptomic domains) to elucidate underlying pathogen–host interaction mechanisms, forecast viral adaptive evolution, and identify novel therapeutic targets such as antimicrobial peptides [20,21]. Given the limitations of conventional bioinformatics in handling the enormous volume of data produced by high-throughput sequencing, AI technologies have emerged as essential tools for modern microbiological research. Specifically, deep learning methods significantly accelerate genomic analysis through rapid alignment against extensive microbial databases, facilitating the detection of previously uncharacterized pathogens, even those with low sequence homology to known species. Notably, during the COVID-19 pandemic, AI played a critical role in the swift identification of SARS-CoV-2 from clinical samples. Beyond detection, AI can also predict critical pathogen characteristics, such as virulence, transmissibility, and host range, based on genomic data, thereby providing crucial support for risk assessment and public health decision-making [22] (Figure 1).

Moreover, the utility of artificial intelligence is especially pronounced in mitigating challenges inherent to sequencing technologies. A notable example lies in addressing the elevated error rates associated with Oxford Nanopore sequencing: both the PrimalSeq wet-lab protocol and the iVar computational pipeline leverage machine learning strategies, utilizing a logistic regression classifier that incorporates mutation frequency and strand bias information to effectively suppress false positive variant calls. This approach offers a tangible framework for enhancing accuracy in long-read data characterized by high error rates. Although not yet a complete solution for data quality control, it underscores the potential of AI-driven methods to significantly improve the reliability of genomic analyses [23].

In summary, artificial intelligence has become an indispensable component across multiple critical domains of high-throughput sequencing-based pathogen research, encompassing antimicrobial resistance gene tracking, data quality control, pathogen identification, and multi-omics integration, thereby offering advanced computational tools for data analysis and predictive modeling in infectious disease studies. Despite these advancements, the clinical translation of AI-powered HTS analysis faces practical hurdles. The dependency on high-quality sequencing data means that performance can be significantly impacted by sample degradation or low pathogen load, which is common in clinical specimens. Furthermore, the computational complexity of some deep learning models may hinder their implementation in time-sensitive diagnostic workflows or resource-limited settings where rapid turnaround is critical. Establishing standardized benchmarks and ensuring computational efficiency are therefore essential next steps.

### 2.2. Outbreak Investigation and Transmission Tracing

AI plays a critical role in HTS-supported outbreak investigation and transmission tracing, significantly enhancing the speed, precision, and intelligence of epidemic response [24]. By integrating genomic data with multisource epidemiological information, AI not only enables efficient pathogen identification and variant monitoring but also demonstrates powerful capabilities in reconstructing transmission chains and predicting evolutionary dynamics [25]. A representative case is a study utilizing nanopore sequencing (MinION) and Hidden Markov Models (HMM), which successfully achieved real-time genomic surveillance and transmission chain tracing of Ebola virus in resource-limited settings. The bioinformatic algorithm processed raw electrical signal data to effectively identify single nucleotide variations (SNVs), significantly enhancing genotyping accuracy under high-error-rate conditions, thereby providing reliable real-time tracing support for field epidemic investigations [26]. Another study developed a CNN-BiLSTM deep learning model for classifying SARS-CoV-2 genomic sequences and identifying key regulatory motifs. Demonstrating significant outperformance over conventional methods with 99.95% accuracy and 100% AUC ROC metrics, this model rapidly distinguishes SARS-CoV-2 from other coronaviruses, highlighting AI’s substantial potential in precisely identifying pathogen-characteristic sequences within high-throughput genomic data [27]. Expanding further to large-scale surveillance practices, a study established a high-throughput SARS-CoV-2 genomic surveillance network, achieving near-real-time viral sequencing and multi-source data integration. This system leveraged cloud computing platforms (e.g., CLIMB) to automate data processing, standardize lineage typing, and integrate clinical information, thereby providing a robust foundation for implementing AI algorithms in transmission chain reconstruction, variant dynamics monitoring, and spatiotemporal modeling, significantly enhancing the precision of outbreak traceability and response capabilities [28].

It is important to note that the effectiveness of these AI-driven phylogenetic approaches is contingent upon sufficient genomic sampling from the outbreak; undersampled outbreaks may lead to incomplete or biased transmission chain reconstruction. In summary, through approaches such as Bayesian inference and machine learning-based clustering, AI facilitates the detection of fine-scale genetic variations and integrates multidimensional metadata, enabling accurate reconstruction of transmission chains and assessment of variant risk. It has thus emerged as an integral component of modern infectious disease surveillance systems and precision public health interventions [29,30].

## 3. AI-Driven Diagnostic Imaging Analysis

High-resolution imaging has revolutionized microbiological and pathological diagnostics by enabling rapid and detailed visualization of pathogenic organisms, thereby significantly enhancing the precision and efficiency of infectious disease diagnosis. The integration of AI, particularly through deep learning-based computer vision algorithms, has further augmented these capabilities, enabling automated, high-throughput analysis of complex diagnostic images with expert-level accuracy.

### 3.1. Digital Microscopy Images

AI-powered diagnostic imaging analysis is progressively transforming conventional malaria detection methods, demonstrating particular advantages in the automated identification and classification of microscope images. AI approaches, primarily deep learning and convolutional neural networks (CNNs), can efficiently process both thin and thick blood smear images to achieve automated detection, classification, and quantification of plasmodium parasites, significantly enhancing diagnostic accuracy and efficiency [31]. Furthermore, AI has not only demonstrated exceptional performance in model accuracy but also proven its substantial value in practical applications. Integrated with smartphone and mobile device-based imaging analysis systems, it provides low-cost, easily deployable malaria screening tools for resource-limited regions, facilitating large-scale disease screening and real-time diagnosis. Moreover, such technology is not limited to malaria but also provides a transferable framework for image-assisted diagnosis of other infectious diseases. Studies have demonstrated that object detection models including YOLOv4, Faster R-CNN, and SSD300 have been successfully applied to the identification and localization of malaria-infected red blood cells in thin blood smear images. Notably, YOLOv4 has demonstrated outstanding performance across multiple evaluation metrics, achieving precision, recall, F1-score, and mean average precision (mAP) of 83%, 95%, 89%, and 93.87%, respectively, at an IoU threshold of 0.5, while exhibiting robust cross-dataset generalization capability. This approach enables end-to-end cell detection and cropping, eliminating the dependency on single-cell image preprocessing, thereby providing a reliable foundation for subsequent parasite species identification and infection stage analysis [32].

A key challenge for deployment, however, lies in the need for consistent slide preparation and imaging conditions, as variations in staining and focus can adversely affect model performance, highlighting a dependency on standardized operational procedures. With the continuous evolution of technology, AI-based microscopic image analysis systems are becoming integral components of modern medical diagnostics. Their breakthroughs in automation, processing efficiency, and accessibility are poised to exert a profound impact on global malaria prevention and control, as well as the rapid diagnosis of other infectious diseases.

### 3.2. Radiological Images

**Figure 2:**
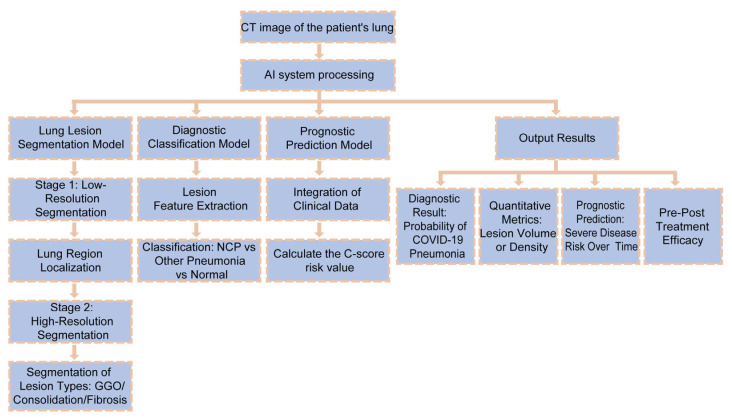
AI-powered diagnostic and prognostic workflow for COVID-19 pneumonia.

The urgent need for rapid and accurate diagnostic technologies during the COVID-19 pandemic has profoundly highlighted the value of AI in medical imaging analysis. Numerous studies have systematically reviewed the application of AI in the radiological diagnosis of COVID-19-induced acute respiratory distress syndrome (ARDS) and pneumonia, spanning the entire workflow from image acquisition and automated segmentation to diagnostic classification and prognostic assessment. Utilizing primarily chest CT, chest X-ray (CXR), and lung ultrasound modalities, AI technologies—leveraging deep learning (e.g., U-Net, CNN, ResNet, DRENet), transfer learning, machine learning, and hybrid models integrating traditional radiomics—have achieved precise segmentation of lung and infected areas, differential diagnosis between COVID-19 and other types of pneumonia, as well as quantitative assessment of disease severity and patient outcomes [33,34,35]. Studies have demonstrated that AI not only efficiently extracts imaging features (such as ground-glass opacity, GGO) and achieves high accuracy in multiple experiments (e.g., AUC up to 0.95, sensitivity and specificity exceeding 90%), but also exhibits excellent interpretability by visualizing critical lesion regions through attention mechanisms, thereby providing reliable decisionsupport for clinicians. Furthermore, AI systems can integrate clinical indicators (e.g., age, comorbidities, laboratory results) to enable early prediction of severe disease risk and assessment of recovery time, thereby supporting optimized allocation of medical resources and stratified patient management. These capabilities demonstrate significant potential for enhancing diagnostic efficiency and reducing healthcare-associated infection risks. Although current challenges such as limited sample sizes, inter-protocol variations in imaging, and model generalizability persist, AI technology has demonstrated significant application value and broad prospects in radiological diagnosis for both COVID-19 and future public health emergencies [36,37,38,39,40,41] (Figure 2).

Clearly, AI has played multifaceted roles in COVID-19 image recognition, quantitative assessment, and prognosis prediction through multimodal and multi-architecture integration strategies, significantly enhancing the comprehensive capabilities of radiological diagnosis. With ongoing algorithm optimization and deeper clinical integration, AI is poised to become an increasingly critical tool in infectious disease control and precision medicine. Notwithstanding the promising results, the generalizability of AI models for medical imaging remains a primary concern. Models trained on data from specific hospitals or scanner protocols often experience performance degradation when applied to external datasets, a phenomenon known as domain shift. This limitation questions the readiness of many reported models for broad clinical deployment without extensive external validation. Additionally, the integration of AI tools into radiologists’ workflow requires careful design to avoid alert fatigue and ensure that AI outputs are actionable, which is an area needing more human-factor studies.

### 3.3. Artificial Intelligence for Nuclear Analysis (AINU)

**Figure 3:**
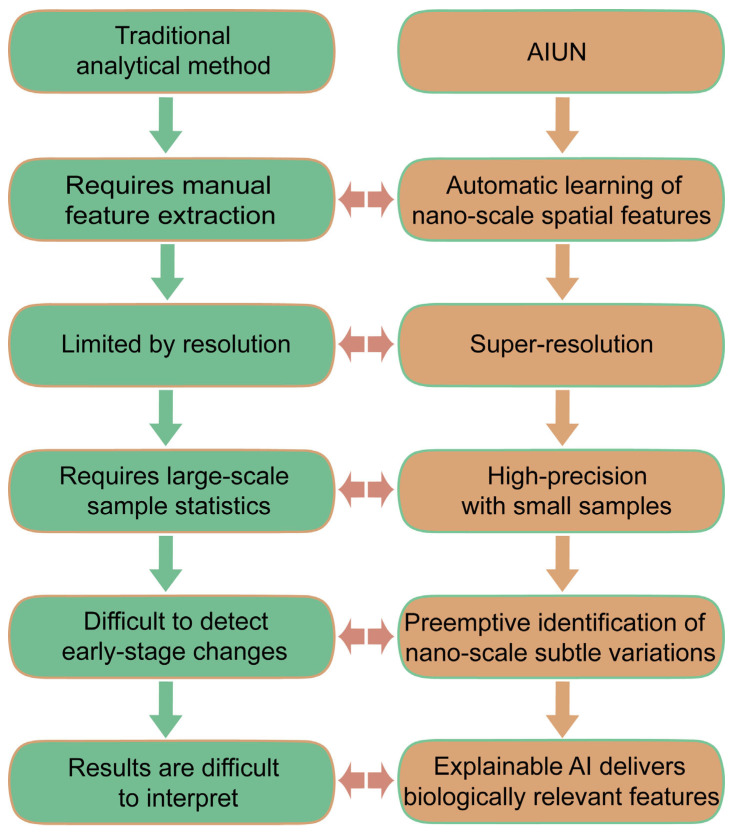
Key advantages workflow: AINU vs. traditional methods.

**Figure 4:**
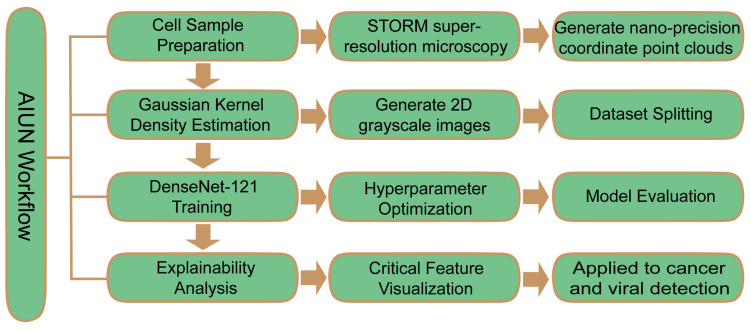
AINU workflow.

A paradigm shift in diagnostic technology is underway, centered on moving from indirect evidence to direct observation of host cell responses. While the previous sections have discussed AI applications that enhance the detection of the pathogen itself (e.g., via sequencing or imaging) or its biomarkers (e.g., via POCT), this section introduces a fundamentally different paradigm. Artificial Intelligence for Nuclear Analysis (AINU) exemplifies a shift towards detecting the infection through the earliest morphological responses of the host cells. Traditional diagnosis of viral infections relies on detecting the pathogen itself or the immune response triggered by infection (Figure 3). In contrast, AINU employs a fundamentally different paradigm by focusing on early-stage, subtle morphological alterations induced within host cell nuclei following viral infection. The core technology of AINU leverages advanced deep learning models to analyze high-dimensional nuclear image data acquired through super-resolution microscopy. This analysis enables the detection of infection-induced abnormalities in key nuclear components, such as alterations in histone modification patterns and spatial distribution and clustering status of RNA polymerase II. This approach demonstrates high sensitivity in identifying infected cells during the very early stages of active viral replication, prior to the emergence of significant cytopathic effects or robust immune responses. By integrating explainable artificial intelligence (XAI) techniques, AINU has identified key nuclear features that discriminate between healthy and infected cell states. This capability provides a novel analytical framework for understanding cellular heterogeneity and elucidating the mechanisms of viral infection [42] (Figure 4). This approach positions AINU as a potential tool for ultra-early diagnosis, operating in a timeframe prior to the accumulation of sufficient pathogen material for conventional detection or the onset of a detectable immune response. Therefore, it complements rather than replaces established methods. While AINU represents a highly specialized and emerging niche, its inclusion here underscores the diversity of AI applications moving beyond traditional paradigms toward ultra-early, morphology-based cellular diagnostics, potentially offering a complementary approach to established methods in future diagnostic workflows [43].

## 4. AI-Enhanced Point-of-Care Testing (POCT)

Syndromic surveillance and multiplex detection capabilities can enhance POCT, while the integration of AI further improves its efficiency, accuracy, and accessibility.

### 4.1. Intelligent Syndromic Surveillance

Driven by the growing demand for precision medicine, infectious disease diagnostics is evolving toward multiplexed, rapid, and intelligent approaches. Within this context, the application of machine learning (ML) in intelligent syndromic detection, that is the simultaneous analysis of multiple biomarkers which has significantly enhanced the accuracy and efficiency of diagnosing infectious diseases. ML technologies have been integrated into various POCT platforms, including lateral flow immunoassays (LFA), vertical flow immunoassays (VFA), nucleic acid amplification tests (NAAT), and imaging sensors, thereby achieving higher sensitivity, specificity, and automation in the detection of a range of infectious diseases such as COVID-19, HIV, malaria, and Lyme disease [44]. For instance, an intelligent detection system based on temporal deep learning architectures (e.g., TIMESAVER algorithm) integrates YOLO, CNN-LSTM, and fully connected networks to enable real-time processing of time-series images from LFA strips. This system completes detection of COVID-19 antigens and influenza A/B viruses within 1–2 min with accuracy exceeding 96%, outperforming 15 min manual interpretation while demonstrating strong versatility across multiple commercial test strips. Notably, it significantly reduces false-negative risks in low viral load samples, making it particularly suitable for time-sensitive scenarios such as emergency departments and field screening [45]. Another representative application is the Rapid Multiplex Molecular Syndromic Panel (RMMSP), which enables simultaneous detection of multiple targets including bacterial, viral, and fungal pathogens as well as antimicrobial resistance genes. Capable of providing results within six hours, this technology significantly accelerates the diagnostic process for critically ill patients, thereby facilitating early targeted antimicrobial therapy, optimizing antibiotic stewardship, and improving clinical outcomes [46].

Such AI-enhanced syndromic detection platforms not only achieve high-throughput, multiplex pathogen detection but also represent a paradigm shift in molecular diagnostics, evolving from traditional pathogen identification tools into intelligent diagnostic assistance systems with clinical interpretation capabilities. The promising performance of AI-enhanced POCT must be balanced against practical constraints. In resource-limited settings, where such tools are most needed, factors like device cost, maintenance requirements, and operational complexity can impede sustainable adoption. Moreover, while AI can improve test interpretation, its effectiveness is ultimately bounded by the underlying analytical performance (e.g., sensitivity/specificity) of the immunoassay or NAAT platform itself. Continuous improvement of both the physical sensing technology and the AI algorithms is therefore indispensable for realizing the full potential of intelligent syndromic surveillance.

### 4.2. AI-Enhanced Diagnostic Devices

Amid the wave of digital transformation reshaping healthcare, AI is emerging as a key driving force behind a paradigm shift in infectious disease diagnostics. AI-enhanced diagnostic devices have demonstrated significant advantages in the detection and surveillance of infectious diseases, particularly by improving diagnostic speed, accuracy, and accessibility. Typically deployed on POCT and wearable sensing platforms, such devices leverage machine learning techniques, including CNNs and deep learning to achieve high-sensitivity and high-specificity detection of a wide range of infectious diseases [47]. In resource-limited settings, AI-enhanced diagnostic devices have demonstrated remarkable success. For instance, in sub-Saharan Africa, portable CNN-based devices achieved 95% sensitivity in detecting malaria parasites. In India, AI-driven sputum analysis combined with telemedicine attained 92% sensitivity for tuberculosis diagnosis while reducing the diagnosis time from weeks to hours. During COVID-19 containment, AI systems enabled rapid triage and risk stratification with high accuracy (AUC-ROC: 89%), significantly shortening isolation periods and improving patient outcomes [48].

AI-enhanced diagnostic technologies have significantly optimized infectious disease diagnosis and control outcomes through high-precision, rapid, and accessible testing capabilities, demonstrating particularly important practical value in resource-limited settings., although long-term sustainability depends on overcoming hurdles related to device durability, connectivity, and continuous user training.

## 5. Available Databases for AI-Driven Infectious Disease Diagnostics

Publicly accessible, high-quality databases serve as the cornerstone for advancing AI in infectious disease diagnostics. These resources, spanning genomics, medical imaging, clinical records, and multi-omics data, provide a rich and diverse foundation for training and validating AI models, significantly enhancing the accuracy and practical utility of diagnostic tools.

### 5.1. Genomic and Sequencing Databases

#### 5.1.1. GISAID (Global Initiative on Sharing All Influenza Data)

Among the publicly available data platforms for training and validating AI models, the Global Initiative on Sharing All Influenza Data (GISAID) plays a pivotal role. GISAID is a critically curated database providing annotated genomic sequences of pathogens like influenza viruses and SARS-CoV-2, coupled with essential metadata such as sampling date and geographic location. This platform is indispensable for developing AI-driven diagnostic and surveillance tools; the large-scale, well-annotated data it provides fuels machine learning models for pathogen identification, classification, and phylogenetic analysis. For instance, leveraging GISAID data, AI algorithms enable dynamic lineage nomenclature, track the evolution of variants, and reconstruct transmission chains, significantly enhancing real-time insights in genomic epidemiology. Thus, GISAID serves as an integral bridge connecting AI technology with practical infectious disease diagnostics [49,50,51,52].

#### 5.1.2. NCBI GenBank and SRA (Sequence Read Archive)

In AI-driven infectious disease diagnostics, public databases such as NCBI GenBank and the Sequence Read Archive (SRA) serve as essential resources. GenBank hosts a vast collection of annotated nucleotide sequences from diverse pathogens, while SRAs raw high-throughput sequencing data from a wide range of clinical and environmental samples. These repositories provide a foundational dataset for training and validating AI models, particularly in tasks such as rapid pathogen identification, resistance gene detection, and metagenomic classification. For instance, k-mer-based classification tools like Kraken leverage reference genomes from GenBank to enable efficient sequence alignment and taxonomic labeling. Meanwhile, the real-time sequencing data available in SRA support AI-powered outbreak investigation and variant surveillance. The accessibility of these datasets significantly advances the application of AI in genomic epidemiology, facilitating precise diagnostics and timely public health responses [53,54,55].

#### 5.1.3. PATRIC (Pathosystems Resource Integration Center)

In AI-driven infectious disease diagnostics, the availability of high-quality, structured databases is crucial for model training and validation. PATRIC (Pathosystems Resource Integration Center) serves as a comprehensive bacterial bioinformatics resource, integrating genomic, transcriptomic, and proteomic data from over 80,000 bacterial genomes, with a strong emphasis on antimicrobial resistance (AMR) metadata and standardized annotations. The platform not only supports comparative genomics but also incorporates machine learning-based AMR phenotype prediction tools, such as k-mer-based AdaBoost classifiers for species like Mycobacterium tuberculosis and Streptococcus pneumoniae, achieving high classification accuracy. By providing antibiotic susceptibility profiles, virulence factor annotations, and host–pathogen interaction data, PATRIC enables robust genotype-phenotype correlation studies, thereby enhancing the interpretability and clinical applicability of AI models in pathogen genomics and resistance prediction [56,57].

### 5.2. Medical Imaging Databases

During the COVID-19 pandemic, the establishment of public imaging datasets significantly advanced the application of AI in infectious disease diagnostics. For instance, several publicly available chest X-ray and CT image datasets (e.g., the COVID-19 Radiography Database) integrate images from patients with COVID-19, other viral pneumonias, and healthy controls, providing a crucial foundation for training and validating deep learning models. These datasets have supported tasks such as automated lesion segmentation, disease classification, and severity assessment, while also facilitating the validation of model generalizability in real-world clinical settings. As highlighted by Wynants et al. (BMJ, 2020) [58] in their systematic review, although many early models suffered from limited sample sizes and high risk of bias, validation studies based on public datasets have offered a vital platform for objective performance evaluation. The open sharing of such data resources has been indispensable for advancing the practical application and standardization of AI-assisted diagnostic imaging—a key focus of this review [58].

Malaria Cell Images Dataset: In the field of malaria diagnosis, publicly available cell image datasets play a crucial role in advancing AI model development. For instance, the malaria cell image dataset hosted by platforms such as the National Institutes of Health (NIH) contains thousands of thin blood smear images collected from hundreds of patients, each meticulously annotated by experts to distinguish between parasitized and uninfected red blood cells. This dataset serves as a benchmark for training and validating convolutional neural networks (CNNs), supporting the development of lightweight models like CFPNet-M tailored for resource-constrained settings. By providing standardized, multi-center image samples, such datasets significantly enhance model generalizability in challenging scenarios including staining variations and cell overlapping, thereby laying a solid data foundation for the clinical translation of AI in malaria microscopic image diagnosis [59,60].

TB Portals Program: In the field of malaria diagnosis, publicly available datasets are crucial for advancing AI model development. For instance, the Malaria Cell Images Dataset, hosted on platforms such as Kaggle and the NIH, contains thousands of thin blood smear images with clear labels indicating parasitized and uninfected red blood cells. This dataset has become a benchmark for training and validating CNN-based models, enabling high-accuracy automated detection of malaria parasites. Its standardization and accessibility significantly promote the application of AI in digital microscopy image analysis, providing a reproducible foundation for algorithm development—particularly relevant to AI-enhanced POCT and intelligent diagnostic imaging discussed in this review [61].

### 5.3. Clinical and Syndromic Surveillance Databases

MIMIC-IV (Medical Information Mart for Intensive Care): The availability of high-quality clinical databases is crucial for the development and validation of AI models in infectious disease diagnostics. MIMIC-IV (Medical Information Mart for Intensive Care) is a large, freely accessible database comprising de-identified electronic health records from intensive care unit patients, which includes comprehensive clinical data such as laboratory results, vital signs, medication records, and diagnostic codes. This resource provides a robust foundation for building AI models aimed at early sepsis prediction, detection of nosocomial infections, and syndromic surveillance. Particularly when integrating multi-source information—such as high-throughput sequencing (HTS), medical imaging, and point-of-care testing (POCT)—MIMIC-IV enables model training and validation in real-world clinical settings, thereby enhancing the generalizability and practical utility of AI-driven diagnostic tools for complex infectious diseases [62,63].

OpenFDA: In terms of publicly available databases, the U.S. Food and Drug Administration’s open data platform, OpenFDA (https://open.fda.gov, accessed on 16 June 2025), offers valuable resources for infectious disease diagnostics. It provides harmonized access via application programming interfaces (APIs) to structured datasets including adverse event reports (FAERS), product labeling (SPL), recall information (RES), and medical device reports (MAUDE). For infectious disease applications, researchers can leverage OpenFDA to access post-market surveillance data related to antiviral drugs, vaccines, or diagnostic devices. AI models can utilize these data for pharmacovigilance tasks—such as mining adverse event signals—assessing diagnostic device performance, or analyzing usage trends during outbreaks. These real-world data enhance the external validation of AI tools and support integrated surveillance of diagnostics and therapeutics in infectious disease management [64].

### 5.4. Multi-Omics and Integrated Databases

In the context of multi-omics data integration for infectious diseases, the Virus Pathogen Database and Analysis Resource serves as a prominent example of a comprehensive platform. ViPR aggregates multi-dimensional viral data—including genomic, proteomic, and immune epitope information—and offers a suite of integrated analysis tools. It particularly supports AI-driven investigations into viral host adaptation, antibody escape prediction, and vaccine design, providing a well-structured and annotated data foundation for in-depth analysis of high-throughput sequencing data. By facilitating combined multi-omics analyses of viral pathogens, ViPR stands as a key public resource that enhances AI-assisted diagnostics and research into pathogen evolution [65,66].

The European Nucleotide Archive (ENA): The European Nucleotide Archive (ENA), a key member of the International Nucleotide Sequence Database Collaboration (INSDC), serves as a vital repository providing high-quality public data for infectious disease diagnostics. ENA integrates large-scale sequencing data from clinical and environmental samples, encompassing raw reads, assembled genomes, and rich spatiotemporal metadata. In AI-driven diagnostic research, ENA supplies essential datasets for pathogen detection, variant tracking, and outbreak investigation. During the COVID-19 pandemic, for instance, ENA’s efficient submission and distribution APIs enabled real-time global sharing and analysis of SARS-CoV-2 genomic data, crucially supporting AI model training and outbreak response. Recent enhancements such as the Pathogens Portal and a tagging system further improve data discoverability and facilitate AI-based identification of pathogenic organisms within complex microbial communities [67,68,69].

These databases represent valuable resources for training, validating, and benchmarking AI models in infectious disease diagnostics. However, challenges remain regarding data standardization, interoperability, and ethical sharing practices. Future efforts should focus on curating multi-institutional, multimodal datasets that are representative of global populations to enhance the generalizability and equity of AI diagnostic tools [70,71,72,73,74,75].

## 6. Challenges and Limitations in AI Diagnostic Technologies

Despite demonstrating substantial potential and achieving remarkable successes in the field of diagnostic assistance, AI continues to face formidable challenges on its path toward large-scale clinical implementation and routine application. Addressing these limitations is critical for realizing the full potential of AI in clinical practice.

### 6.1. Economic and Administrative Burden

First, the adoption of AI in medical fields imposes significant economic and administrative burdens on healthcare institutions. To ensure patient safety, medical AI systems must undergo rigorous regulatory approval processes demonstrating safety, efficacy, and transparency before clinical deployment. This process is often protracted and cost-prohibitive, involving large-scale clinical trials and complex regulatory documentation, thereby imposing substantial financial strain on both development institutions and healthcare facilities [76]. Beyond initial deployment, AI systems require continuous investment, including iterative data integration, software updates, and hardware maintenance. These systems demand specialized personnel in medical informatics, data science, and IT support for ongoing system maintenance, updates, and user training, constituting persistent economic and administrative burdens for healthcare institutions [77,78].

### 6.2. Regulatory and Clinical Integration Barriers

Moreover, the regulatory landscape for medical AI is rapidly evolving worldwide, and stringent regulations impede the progression from development and validation to full integration into healthcare systems; this poses significant challenges for medical institutions [79,80,81,82]. Furthermore, AI tools face linguistic and cultural application constraints. When deployed in diverse linguistic environments or among populations with unique cultural health beliefs, their performance may significantly decline, necessitating localization adjustments that require additional resources and time investment [83,84]. Additionally, a critical challenge lies in effectively communicating the complex numerical outputs of AI models to physicians in an accurate, intuitive, and clinically meaningful manner, while seamlessly integrating them into clinical decision-making processes. Establishing efficient human–AI interaction interfaces is imperative to ensure that AI-generated recommendations are correctly interpreted and appropriately adopted in practice [85].

### 6.3. Data Bottlenecks and Model Limitations

In complex scenarios such as medical imaging, AI must rely on vast amounts of data for deep training and iterative algorithm optimization to achieve accurate and reliable analysis [86]. However, large-scale, well-annotated datasets remain scarce and costly to generate. Discrepancies in diagnostic criteria across regions and stringent patient privacy protection requirements exacerbate difficulties in data integration and sharing, ultimately severely impeding the deep application and widespread adoption of AI in medical imaging [87].

Annotation scarcity and the difficulty of generating large-scale, high-quality labeled datasets remain among the most critical barriers to clinical AI implementation. To address this, the research community is actively developing innovative strategies. Self-supervised learning (SSL) offers a promising avenue by pre-training models on vast amounts of unlabeled data (e.g., raw medical images or genomic sequences) to learn meaningful representations before fine-tuning on limited labeled datasets. For instance, models pre-trained with SSL on large chest CT archives have shown improved performance in downstream tasks like COVID-19 classification with minimal labeled examples [88,89]. Semi-supervised learning and active learning frameworks can strategically select the most informative samples for expert annotation, maximizing model performance gains while minimizing labeling effort [90,91,92]. Furthermore, synthetic data generation techniques, particularly generative adversarial networks (GANs), can create realistic, annotated medical data to augment small training sets, thereby improving model robustness and helping to address class imbalance [93,94]. However, ensuring the clinical fidelity and avoiding bias propagation in synthetic data remain challenges. Finally, domain adaptation and generalization methods are crucial for enhancing model robustness across different hospitals, scanner protocols, and patient populations. These techniques aim to learn invariant features that perform well on data distributions different from the training set, which is essential for real-world deployment [95,96].

While these approaches show considerable promise, their integration into standardized diagnostic workflows and regulatory acceptance are ongoing endeavors. Future work should focus on benchmarking these methods in large-scale, multi-center studies specific to infectious disease diagnostics to validate their practical utility.

## 7. Summary and Future Directions

### 7.1. Summary

This review systematically examines the rapid advancement of AI in infectious disease diagnostics and its multifaceted clinical utility. In the domain of high-throughput sequencing, AI has demonstrated transformative capabilities in pathogen identification, characterization, and real-time surveillance of outbreaks and transmission patterns. In the realm of image analysis, AI enables automated detection and quantification of pathogens in digital microscopy images while enhancing diagnostic accuracy for various infectious diseases through radiological imaging. For point-of-care testing, AI augments syndromic surveillance platforms and facilitates the development of intelligent, field-deployable diagnostic devices.

Despite these advancements, significant challenges persist in data availability, model generalizability, economic sustainability, and clinical integration. Addressing these limitations requires collaborative efforts among clinicians, microbiologists, computational scientists, and policymakers. Future directions should focus on developing more robust and adaptable AI systems, establishing standardized datasets and evaluation frameworks, and creating streamlined regulatory pathways for AI-based infectious disease diagnostics. Through this multidisciplinary approach, AI can realize its full potential to revolutionize infectious disease diagnosis and management, ultimately enhancing global health security.

### 7.2. Future Directions

Despite the promising advances outlined in this review, the full integration of AI into routine infectious disease diagnostics requires addressing several persistent challenges. Future research and development efforts should therefore prioritize a number of key directions to bridge the gap between technological potential and clinical utility.

First, overcoming data-related bottlenecks is paramount. Efforts should focus on the development and adoption of standardized, large-scale, and well-annotated multimodal datasets that are representative of diverse populations and settings. To mitigate the high cost of manual annotation, exploring advanced techniques such as self-supervised learning, federated learning for privacy-preserving collaborative model training, and synthetic data generation holds significant promise for enhancing model robustness and generalizability.

Second, the next generation of AI models must be designed with clinical translation in mind. This involves creating more interpretable and trustworthy systems through the integration of explainable AI (XAI) principles, enabling clinicians to understand the rationale behind AI-generated insights. Furthermore, developing lightweight and computationally efficient algorithms will be critical for deploying AI models on mobile or point-of-care devices in resource-limited environments.

Finally, successful clinical integration will depend on establishing streamlined regulatory pathways and fostering effective human-AI collaboration. Prospective, multi-center clinical validation studies are urgently needed to generate robust evidence of efficacy and cost-effectiveness. Simultaneously, research should focus on designing intuitive user interfaces and clinical workflows that seamlessly integrate AI outputs into decision-making processes, ultimately supporting rather than replacing clinical expertise.

By addressing these priorities through collaborative efforts among clinicians, researchers, regulatory bodies, and industry partners, AI can evolve from a powerful analytical tool into a dependable ally in the global fight against infectious diseases.

## Data Availability

No new data were created or analyzed in this study. Data sharing is not applicable to this article.

## Abbreviations

The following abbreviations are used in this manuscript:

AI	Artificial Intelligence
AINU	Artificial Intelligence of the Nucleus
CBCT	Cone-Beam Computed Tomography
ML	Machine Learning
HTS	High-Throughput Sequencing
POCT	Point-of-Care Testing
SVM	Support Vector Machines
HMM	Hidden Markov Models
SNVs	Single-Nucleotide Variations
mAP	Mean Average Precision
ARDS	Acute Respiratory Distress Syndrome
CXR	Chest X-ray
XAI	Explainable Artificial Intelligence
LFA	Lateral Flow immunoassays
VFA	Vertical Flow immunoassays
NAAT	Nucleic Acid Amplification Tests
CNN	Convolutional Neural Network(s)
BiLSTM	Bidirectional Long Short-Term Memory
GANs	Generative Adversarial Networks
SSL	Self-Supervised Learning
RMMSP	Rapid Multiplex Molecular Syndromic Panel
